# Effect of the Nature of Metal Ions and the Type of Solvent on the Mechanical, Self-Healing and Conductive Properties of Poly(AA-Co-AAm) Gels

**DOI:** 10.3390/gels12070565

**Published:** 2026-06-26

**Authors:** Arsenii Fateev, Yulia Katina, Mikhail Litvinov, Vera Sitnikova, Aleksandr Podshivalov

**Affiliations:** Center for Chemical Engineering, ITMO University, Kronverksky Pr. 49, 197101 Saint Petersburg, Russiav.e.sitnikova@gmail.com (V.S.); podshivalov@itmo.ru (A.P.)

**Keywords:** hydrogel, PEG-gel, self-healing, DMA, hysteretic behavior, ions of polyvalent metals

## Abstract

Composite hydrogel based on acrylic acid and acrylamide, modified with metal ions (*Ni*^2+^, *Al*^3+^, *Fe*^2+^, *Fe*^3+^) with concentration 0.3 wt%, were synthesized in water or polyethylene glycol (with a molecular weight of 400 Da) at three monomer ratios (7/3, 1/1, 3/7). Dynamic mechanical analysis shows that the equilibrium modulus of elasticity ($G_{e}$) of unmodified hydrogels increases with acrylamide content due to higher crosslinking density ($n_{e}$) and smaller cell size. ${AlCl}_{3}$ or ${NiCl}_{2}$ strengthen the structure ($G_{e}$ increases +53.5% in a 1/1 ratio), while iron salts cause softening (decreases to 90% when using ${FeC}_{2}O_{4}$). Partial replacement of polyethylene glycol reduces the elasticity but when using ${AlCl}_{3}$ happens synergistic increase $n_{e}$ 1.9 times in the ratio 3/7. The self-healing efficiency reaches ~100% for ${FeCl}_{3}$ in PEG gel in a ratio of 1/1 and 72.1% for ${Fe({NH}_{4})}_{2}{\left({SO}_{4}\right)}_{2}$ hydrogel in 3/7. The electrical conductivity of hydrogels increases in the range of ${Al}^{3+}>{Ni}^{2+}>{Fe}^{3+}$, while matrix based on polyethylene glycol reduces the conductivity by an order of magnitude. For ${Ni}^{2+}$-containing samples, pinched hysteresis loops are observed in both water and polyethylene glycol. In contrast, ${Al}^{3+}$ causes rapid passivation in the water matrix, while in the matrix based on polyethylene glycol, the current–voltage characteristics follow ohmic behavior. The results demonstrate the possibility of directional regulation of the mechanical, electrical, and self-healing efficiency of hydrogels by selecting the ratio of monomers, the nature of the ion modifier, and the type of solvent.

## 1. Introduction

Hydrogels are three-dimensional polymer meshes capable of holding significant volumes of water, which leads to their widespread use in biomedicine [1,2], soft robotics [3,4], sensors [5,6,7] and electronics [8,9,10]. Among the various systems, copolymers of acrylic acid (AA) and acrylamide (AAm) are of particular interest, since the combination of carboxyl and amide groups allows for varying hydrophilicity, pH sensitivity, and mechanical properties [11,12,13]. However, achieving a balance between strength, elasticity, self-healing ability, and electrical conductivity remains a key challenge, as these characteristics are often mutually exclusive in classical covalent meshes [14,15,16].

One of the promising approaches to regulating the properties of hydrogels is the introduction of metal ions. The coordination interaction between the cations (e.g., ${Ni}^{2+},\;{Al}^{3+},\;{Fe}^{3+},\;{Fe}^{2+}$) and carboxylate groups allow you to create additional physical crosslinking [17,18], which can lead to reinforcement of the grid and to the emergence of reversible linkages necessary for self-healing [19]. At the same time, the nature of the anion, the redox properties of iron ions, and the ratio of AA⁄AAm monomers critically affect the structure of the polymer matrix during radical copolymerization [20,21].

It should be noted that the practical application of water-based hydrogels is complicated by their instability during storage and operation. Thus, evaporation of water leads to shrinkage of the mesh, a sharp deterioration in mechanical properties, a decrease in ionic conductivity and loss of self-healing ability. Airtight conditions or constant humidity support are required to prevent drying, which significantly limits technological flexibility. One of the approaches to solving this problem is the partial replacement of water with organic solvents (such as glycerin, ethylene glycol, and sorbitol) [22,23,24]. In our work, polyethylene glycol (PEG) was used to replace the solvent, as it is non-volatile, biocompatible, capable of retaining moisture and plasticizing the polymer matrix [25]. This modification allows not only to stabilize properties over time without strict environmental control, but also to change the topology of the grid due to competition for hydrogen bonds and coordination of metal ions [26]. However, replacing some of the water with PEG can change the mobility of the chains, compete for coordination with ions, and affect the morphology of the gel [27].

This work presents the synthesis and comprehensive analysis of composite gels based on P(AA-co-AAm), modified salts ${Ni}^{2+},\;{Al}^{3+},\;{Fe}^{3+}and\;{Fe}^{2+}$ at three ratios of monomers (7/3, 1/1, 3/7) in the aquatic environment and in the environment of low molecular weight PEG. Using dynamic mechanical analysis (DMA), electrical conductivity measurements, and self-healing tests, we investigate the effect of ion modification on the equilibrium accumulation modulus ($G_{e}$), crosslinking density ($n_{e}$), mesh size (ξ), and hysteretic behavior. The aim of our work is to establish a correlation between the chemical composition, the topology of the grid and the functional properties (mechanical, reducing, electrical) and to determine the optimal combinations of “matrix—modifier—solvent” to create hydrogels with specified characteristics.

## 2. Results and Discussion

### 2.1. Structure and Water Content of AA-Co-AAm Hydrogels and PEG-Gels

**Figure 1:**
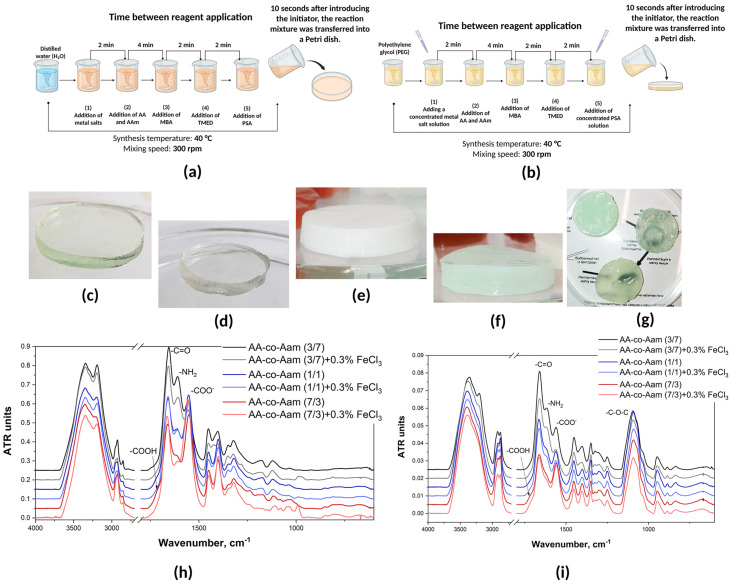
Schematic representation of the fabrication process for water-based hydrogels (**a**) and PEG-based organogels (**b**). Synthesized samples of hydrogel containing Ni^2+^ (NiCl_2_) (**c**) and unmodified hydrogel (**d**); Unmodified PEG gel (**e**) and PEG gel containing Ni^2+^ (NiCl_2_) (**f**); comparison of PEG gel containing Ni^2+^ (NiCl_2_) with different monomer ratios (AA/AAm) 7/3, 1/1 and 3/7 (from transparent to less transparent, respectively) (**g**). ATR-FTIR spectra of hydrogels (**h**) and PEG gels (**i**) with different monomer ratios modified with NiCl_2_ salt 0.3% wt.

In accordance with the procedure described in Section 4.2 and Section 4.3, a series of polymer gels based on acrylic acid (AA) and acrylamide (AAm) with varying molar ratios of monomers was synthesized. Figure 1 shows the synthesis schemes of hydrogel and organogel, as well as the resulting samples.

Since polyethylene glycol is a good solvent for AA and AAm, replacing water with PEG-400 in the standard hydrogel synthesis method results in transparent, soft, elastic PEG gels that do not show signs of phase separation (Figure 1).

To confirm the structure of the synthesized hydrogels and PEG gels, their IR spectra are presented (Figure 1h,i). Figure 1h shows the IR spectra of hydrogels with different monomer compositions, as well as gels modified with ${FeCl}_{3}$ salt.

The FTIR spectra of the presented hydrogels have, depending on the monomer ratio, regularly changing ratios of the intensities of the absorption bands of the -C=O carbonyl groups (1660 cm^−1^) of the AA monomer and the -COO^−^ carboxylate groups (1550 cm^−1^) of the AA monomer [28]. When modifying the hydrogel with FeCl_3_ salt, it can be seen from the change in the wavenumber of the corresponding absorption bands that complexation with the hydrogel matrix occurs due to the -COOH bonds of the non-neutralized part of AA (1713 cm^−1^), -NH_2_ of the AAm groups (1612 cm^−1^) and the carboxylate functional groups (1550 cm^−1^) of the neutralized part of the AA monomers [29].

In PEG gels (Figure 1i), in addition to the regular appearance of absorption bands characteristic of PEG (-C-O-C at 1094 cm^−1^), a smaller number of carboxylate groups -COO- are also observed compared to the hydrogel (1550 cm^−1^), but the nature of the binding of iron ions is the same. The shift of the main absorption band of PEG C-O-C from 1094 to 1090 cm^−1^ also suggests an interaction between the iron salt and PEG [30,31].

**Figure 2:**
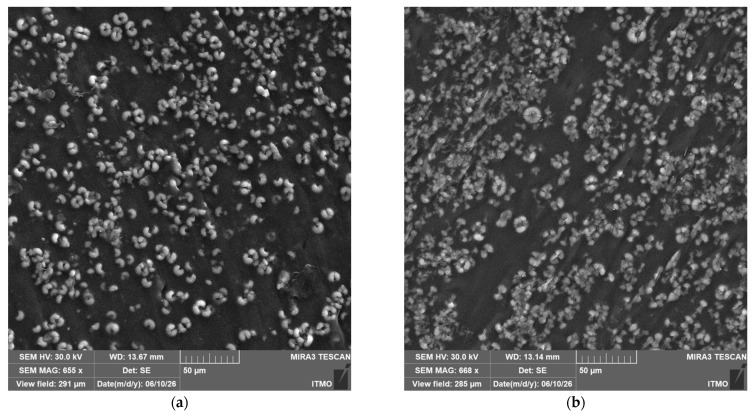

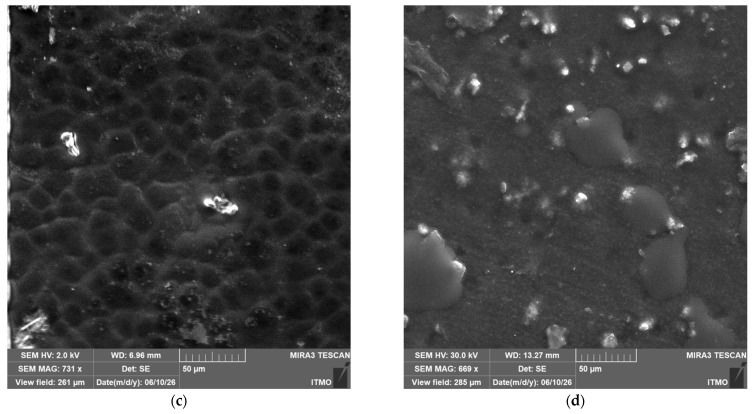
Composite SEM micrographs of hydrogels and PEG gels (scale = 50 microns): (**a**) pure hydrogel; (**b**) hydrogel with Ni^2+^ (0.3 wt%); (**c**) pure PEG gel; (**d**) PEG gel with Ni^2+^ (0.3 wt%).

**Figure 3:**
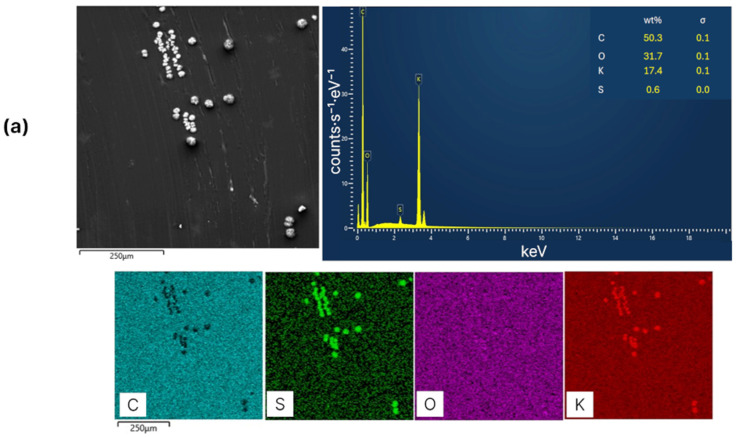

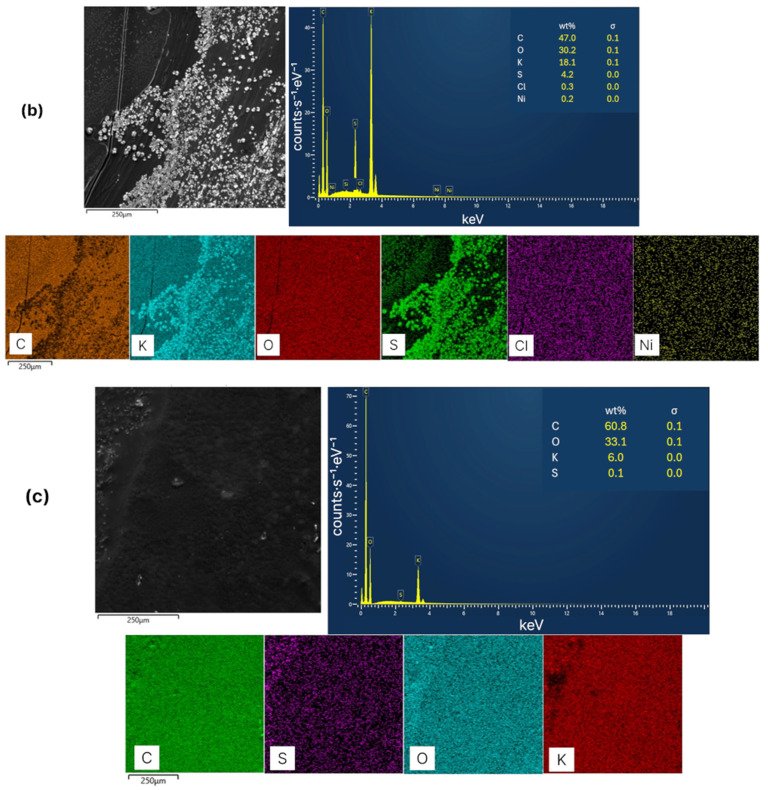

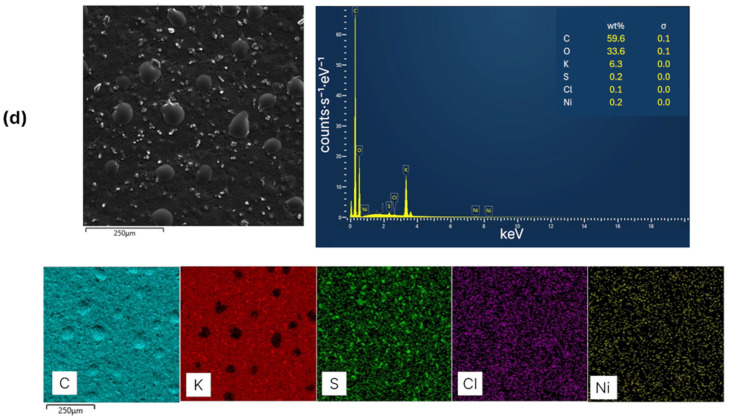
SEM-EDX mapping of elemental distribution on the AA-co-AAm hydrogels and PEG-gels: (**a**) pure hydrogel; (**b**) hydrogel with Ni^2+^ (0.3 wt%); (**c**) pure PEG-gel; (**d**) PEG-gel with Ni^2+^ (0.3 wt%).

Figure 2 and Figure 3 show SEM micrographs and the results of an elemental chemical analysis of hydrogels and PEG gels with a monomer ratio of AA/AAm = 1/1, as well as NiCl_2_-modified gels. According to EDX mapping data (Figure 3b,c), Ni^2+^ ions are distributed homogeneously over the gel matrix, without forming visible aggregates, which is typical for other modifying additives under study.

Micrographs of the unmodified hydrogel (Figure 2a) show surface inhomogeneities that can be identified as crystalline inclusions. Elemental analysis (Figure 3a) identifies them as potassium sulfate (K_2_SO_4_), a residual by-product of the synthesis route. Their formation is due to the presence of K^+^ cations (from potassium acrylate produced by acid neutralization) and sulfate anions, which originate from decomposition of the initiator ammonium sulfate.

The purification step was not applied to the main samples presented in this study for two reasons: (i) crystallization occurs during the final stage of drying when the electrolyte reaches its solubility limit in the concentrated aqueous solution and does not interfere with formation of the internal gel network, and (ii) the presence of these inclusions allows observation of the structuring effect of ions in the native state.

It is noteworthy that for PEG gels, K_2_SO_4_ salting is not observed on the surface. This is probably due to the complex activity of PEG ethylene oxide chains with respect to K^+^ cations, which reduces their mobility and prevents crystal nucleation. At the same time, Ni^2+^ ions exhibit high stability of retention by functional groups (carboxylate ions and amide groups) in both hydrogel and PEG matrices, maintaining the molecular level of dispersion even in the completely dried state, which is confirmed by the absence of phase separation on EDX maps.

The presence of K_2_SO_4_ crystals on the surface does not significantly affect the bulk mechanical properties or resistive-switching characteristics, because the crystals are confined to the outer surface (as shown in the micrographs) and do not penetrate the polymer matrix. Internal conductivity and deformation are governed by polymer–ion interactions within the gel volume, which are not influenced by these surface crystalline deposits.

The water content of hydrogels and PEG gels was determined using TGA and DSC. Table 1 shows the water content of the gels, as well as the proportion of free and semi-freezable water, and strongly bound water.

As can be seen from Table 1, the total water content in hydrogels and PEG gels with different monomer compositions and different types of modifiers is comparable, which allows them to be further studied using the rheological method.

The mass of freezing and non-freezing bound water per gram of dry hydrogel, calculated using formula (5), increases slightly in salt-modified hydrogels.

When replacing the main part of water with PEG-400, DSC analysis shows that no free or semi-freezable water remains in the PEG gels (no melting peak in the DSC curve), and the amount of strongly bound water in the PEG gel depends on the proportion of AA monomers. The introduction of salt as a modifier increases the proportion of strongly bound water, as they are introduced during synthesis as concentrated aqueous solutions.

Furthermore, DSC analysis shows that PEG gels exhibit much lower T_g_ temperatures compared to hydrogels.

### 2.2. Mechanical Performance of AA-Co-AAm Hydrogels and PEG-Gels

**Figure 4:**
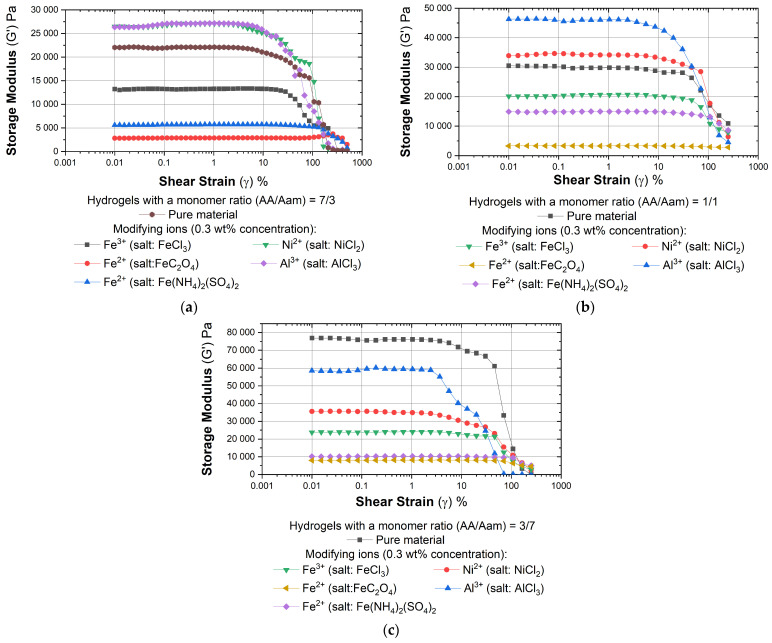
Dependence of the shear modulus ($G^{\prime }$) on shear strain (γ) for hydrogels based on the AA-co-AAm copolymer for pure material (1), with a content of 0.3 wt.% ${Fe}^{3+}({FeCl}_{3})$ (2), with a content of 0.3 wt.% ${Fe}^{2+}({FeC}_{2}O_{4})$ (3), with a content of 0.3 wt.% ${Fe}^{2+}({Fe({NH}_{4})}_{2}{\left({SO}_{4}\right)}_{2})$ (4), with a content of 0.3 wt.% ${Ni}^{2+}({NiCl}_{2})$ (5), with a content of 0.3 wt.% ${Al}^{3+}({AlCl}_{3})$ (6) for ratios of monomers AA/AAm 7/3 (**a**), 1/1 (**b**), 3/7 (**c**). Measured at 10 Hz and 25 °C.

**Figure 5:**
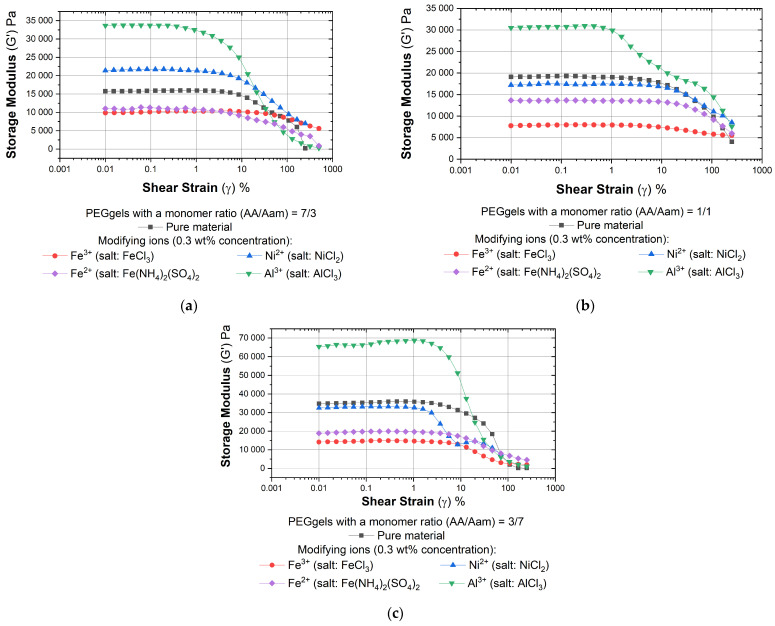
Dependence of the shear modulus ($G^{\prime }$) on shear strain (γ) for PEG-gels based on the AA-co-AAm copolymer for pure material (1), with a content of 0.3 wt.% ${Fe}^{3+}({FeCl}_{3})$ (2), with a content of 0.3 wt.% ${Fe}^{2+}({Fe({NH}_{4})}_{2}{\left({SO}_{4}\right)}_{2})$ (3), with a content of 0.3 wt.% ${Ni}^{2+}({NiCl}_{2})$ (4), with a content of 0.3 wt.% ${Al}^{3+}({AlCl}_{3})$ (5) for ratios of monomers AA/AAm 7/3 (**a**), 1/1 (**b**), 3/7 (**c**). Measured at 10 Hz and 25 °C.

To evaluate the effect of ionic modification on the elasticity and topology of the polymer network, hydrogels (Figure 4) and PEG gels (Figure 5) synthesized at three different ratios of acrylic acid (neutralized with 80% potassium) and acrylamide (7/3, 1/1, 3/7) were studied by DMA. The concentration of the introduced modifiers by metal ion was 0.3 wt.% of the total monomers.

The equilibrium storage modulus ($G_{e}$), the crosslinking density ($n_{e}$) and average mesh size (ξ) were considered key parameters characterizing the spatial structure. The obtained data are systematized in Table 2.

Analysis of the parameters of pure (unmodified) hydrogel samples reveals a clear correlation between the copolymer composition and the characteristics of the polymer network. When moving from a composition with a higher proportion of acrylic acid (7/3) to a composition with a predominance of acrylamide (3/7), a monotonic increase in the equilibrium storage modulus ($G_{e}$) is observed from 22.04 kPa to 76.23 kPa, which corresponds to an increase of more than 3.4 times. The crosslinking density ($n_{e}$) increases from 8.89 mol/m^3^ to 30.75 mol/m^3^, and the average distance between network nodes (ξ) decreases consistently from 5.72 nm to 3.78 nm.

Replacing most of the water with PEG in the gel leads to a decrease in the elastic modulus and crosslinking density across the entire range of monomer ratios. For example, for the 7/3 composition, the $G_{e}\;$ and $n_{e}$ values decrease from 22.040 kPa and 8.89 mol/m^3^ (without PEG) to 15.869 kPa and 6.40 mol/m^3^ (with PEG). A similar trend persists for the 1/1 and 3/7 compositions. This suggests that the high PEG content (70%) acts as a plasticizer or network extender, increasing free volume and reducing the effective crosslinking density by shielding interchain interactions.

The introduction of metal salts has a multidirectional effect on the structural parameters of the gels, with the nature of the effect determined by the cation type and monomer ratio. This effect differs significantly between the hydrogel and the PEG gel. As the values of the linear viscoelastic range (LVE-R) in Table 3 show, modification of hydrogels with nickel chloride and aluminum chloride salts for all monomer ratios leads to the formation of more elastic hydrogels (the LVE-R limit decreases relative to the unmodified hydrogel). For PEG gels, a decrease in the LVE-R limits is characteristic only of gels modified with AlCl_3_ salts.

### 2.3. Self-Healing Efficiency of Hydrogels and PEG-Gels

**Figure 6:**
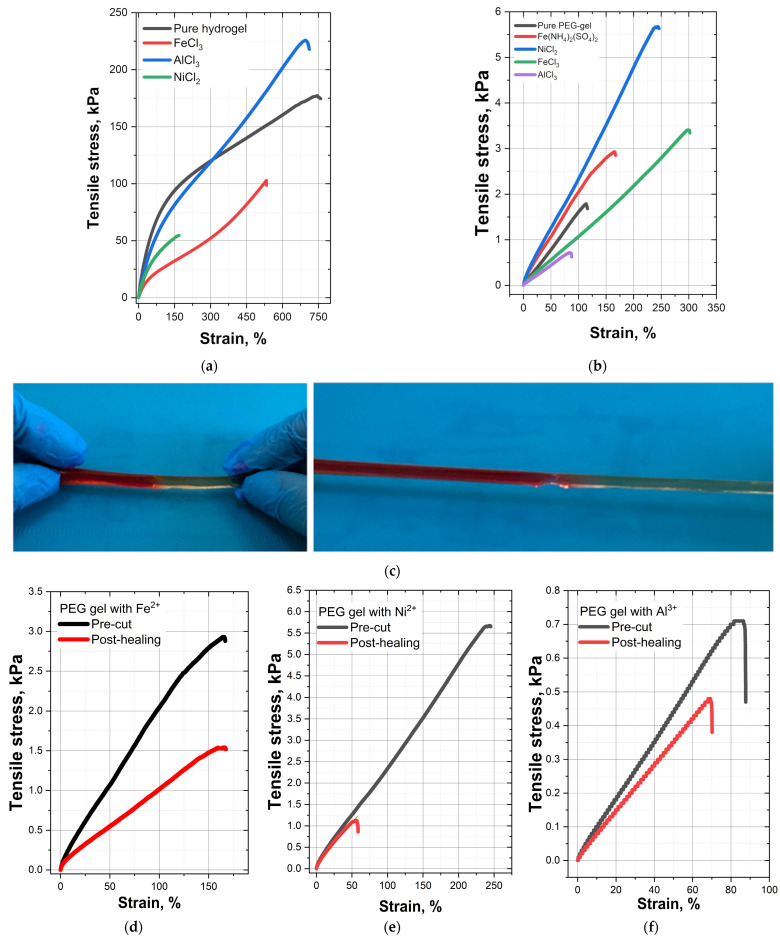

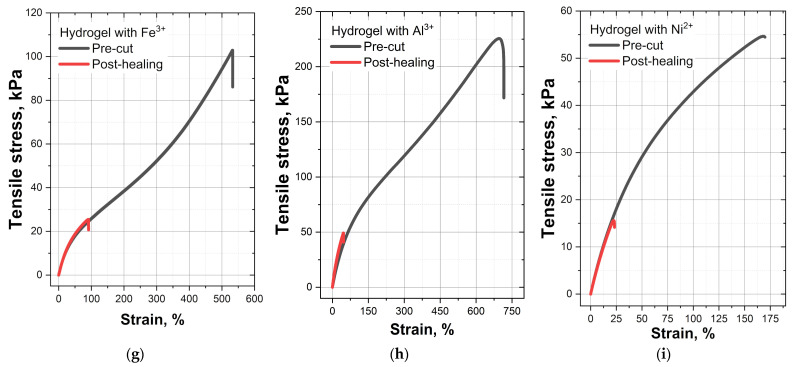
Tensile load–strain curves of P(AA-co-AAm) hydrogels (**a**) and PEG-gels (**b**) with a monomer weight ratio of AA/AAm = 3/7, modified with 0.3 wt% of different metal salts: unmodified, AlCl_3_, NiCl_2_, FeCl_3_, and Fe(NH_4_)_2_(SO_4_)_2_. Curves are truncated after the maximum load to show the pre-failure region; (**c**) Efficiency of self-healing of polymer material modified with iron ions (FeCl_3_). (**d**–**f**) Tensile load–strain curves for PEG-gels modified with Fe(NH_4_)_2_(SO_4_)_2_ (**d**), NiCl_2_ (**e**), and AlCl_3_ (**f**), showing the comparison between intact (original) and healed samples after 3 days of exposure to ~90% humidity. (**d**–**f**) Tensile load–strain curves for hydrogels modified with FeCl_3_ (**g**), NiCl_2_ (**h**), and AlCl_3_ (**i**), showing the comparison between intact (original) and healed samples after 3 days of exposure to ~90% humidity.

Self-healing efficiency ($E_{self}$, %) was assessed using the restoration of mechanical properties after polymer material failure (Figure 6). The results are presented in Table 3.

The stretching curves (Figure 6a,b) show that the mechanical behavior of hydrogels and PEG gels differs significantly. Hydrogels are characterized by significantly higher strength values: for example, the maximum stress reaches ~226 kPa (AlCl_3_), ~102 kPa (FeCl_3_) and ~54 kPa (NiCl_2_), whereas for PEG gels these values do not exceed ~5.7 kPa. In most cases, hydrogels are also superior to PEG gels in terms of fracture deformation: pure hydrogel is ~755% versus ~112%, with AlCl_3_—~700% versus ~81%, with FeCl_3_—~535% versus ~297%. NiCl_2_ is an exception: here the hydrogel undergoes deformation of only ~162%, while the corresponding PEG gel reaches ~240% and this difference indicates a specific structure-directing effect of nickel ions in the hydrophilic matrix.

Figure 6d–f show the stretching curves of the initial (whole) and recovered samples of PEG gels with the ratio of monomers AK/AA = 3/7, modified with AlCl_3_, NiCl_2_, and Fe(NH_4_)_2_(SO_4_)_2_. The analysis of deformation at break allows us to identify two types of mechanical behavior after self-healing. For an iron-containing gel, the most pronounced increase in stiffness is observed, which manifests itself in a significant decrease in fracture deformation. For the sample with Al^3+^, this effect is much weaker, whereas for Ni^2+^, deformation changes are practically not recorded. A similar pattern is typical for other systems.

Even though the iron-containing gel shows the greatest increase in brittleness (loss of deformability), its initial modulus of elasticity drops by almost half, which is significantly stronger than in systems with aluminum and nickel. This indicates that in the process of self-healing, a deep restructuring of the mesh occurs under the action of moisture (~90%): some of the original ordered interchain interactions are irreversibly lost, which reduces the initial resistance to deformation. At the same time, Fe^3+^ ions with high coordination capacity form new, additional coordination nodes with carboxylate and amide groups, which limit the mobility of chains under heavy loads and cause premature destruction (the “over-stitching” effect described for metal-coordinated hydrogels [32]).

It is important to emphasize that this effect is not universal. In systems with Al^3+^ and Ni^2+^, where coordination binding proceeds differently or has a lesser effect on the topology of the grid, no such rearrangement occurs: the modulus changes insignificantly. Consequently, the increase in stiffness after self-healing is specific mainly for Fe-containing compounds and directly correlates with their ability to redistribute ion nodes during the repair process.

On the contrary, for hydrogel systems, this effect is completely absent for all the ions studied. Even for Fe^3+^, which showed the most pronounced increase in stiffness in the PEG matrix, there was no increase in stiffness in the hydrogel. Therefore, the effect of excessive crosslinking is characteristic of the combination of Fe and PEG matrix and is not observed in hydrogels without PEG or in systems with Al^3+^ and Ni^2+^.

In the unmodified state, the self-healing efficiency for the hydrogel with the AA/AA ratio = 7/3 is absent (0%). The introduction of modifiers makes it possible to achieve positive values: the maximum efficiency was recorded for the sample with Fe(NH_4_)_2_(SO_4_)_2_—51.47%, while FeCl_3_ and AlCl_3_ show low values (5.36% and 7.12%, respectively), and NiCl_2_ occupies an intermediate position (31.41%). For the equimolar composition, pure hydrogel demonstrates an efficiency of 18.88%. Modification with salts makes it possible to significantly enhance the self-healing efficiency: the maximum value is achieved for FeCl_3_ (76.76%), for Fe(NH_4_)_2_(SO_4_)_2_—65.45%, and for NiCl_2_—60.25%. The lowest self-healing efficiency is achieved with the use of AlCl_3_ salt (23.85%). With an excess of acrylamide (ratio 3/7), the pure hydrogel shows an efficiency of approximately 26.02%. Modification with Fe(NH_4_)_2_(SO_4_)_2_ leads to a significant increase in the indicator to 72.12%, while NiCl_2_ and FeCl_3_ reduce the efficiency (20.28% and 17.32%, respectively), and AlCl_3_ demonstrates an extremely low value (6.44%).

For pure PEG gel with a monomer ratio of AA/AAm = 7/3, the self-healing efficiency is about 49.08%. Modification with FeCl_3_ and Fe(NH_4_)_2_(SO_4_)_2_ allows for a slight increase in this indicator (54.74% and 48.54%, respectively), while NiCl_2_ and AlCl_3_ lead to a decrease in this indicator (28.53% and 24.62%, respectively).

At equimolar composition, pure PEG gel demonstrates a self-healing efficiency of 27.24%. Modification with FeCl_3_ and NiCl_2_ salts leads to almost complete recovery (~100% and 90.4%, respectively), while modification with Fe(NH_4_)_2_(SO_4_)_2_ and AlCl_3_ salts shows moderate self-healing values (55.21% and 46.14%, respectively).

The record-high self-healing efficiency of the PEG gel modified with FeCl_3_ salt (~100%) is achieved with the most significant decrease in the crosslinking density relative to the pure sample ($n_{e}$ decreases from 7.72 to 3.19 mol/m^3^, ξ increases from 5.99 to 8.04 nm). Apparently, the formation of an extremely loose structure ensures maximum chain mobility. The high self-healing efficiency of the PEG gel modified with NiCl_2_ salt (90.4%) is also accompanied by a decrease in the crosslinking density ($n_{e}$ = 7.02 mol/m^3^) compared to the pure sample. For PEG gels modified with AlCl_3_ salt, on the contrary, an increase in the crosslinking density to 12.39 mol/m^3^ is observed, which explains the lower self-healing efficiency (46.14%).

A pure PEG gel with an AA-AAm monomer ratio of 3/7 exhibits an efficiency of approximately 18.98%. Modification with Fe(NH_4_)_2_(SO_4_)_2_ and AlCl_3_ salts enables high self-healing efficiency of ~100% and 79.98%, respectively. The introduction of FeCl_3_ and NiCl_2_ salts into the PEG gel also increases the self-healing efficiency, but to more moderate values (66.24% and 62.24%).

### 2.4. Electrical Conductivity Properties of Hydrogels and PEG-Gels

**Figure 7:**
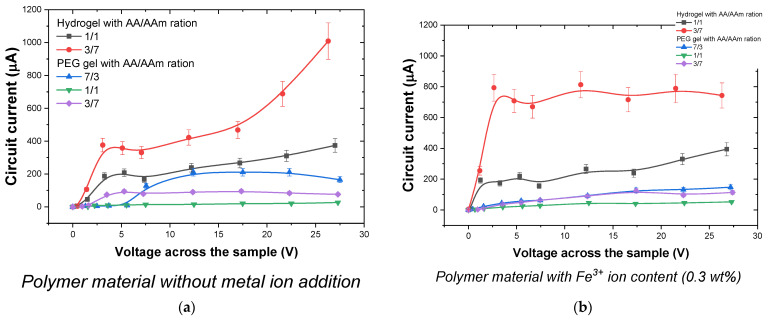

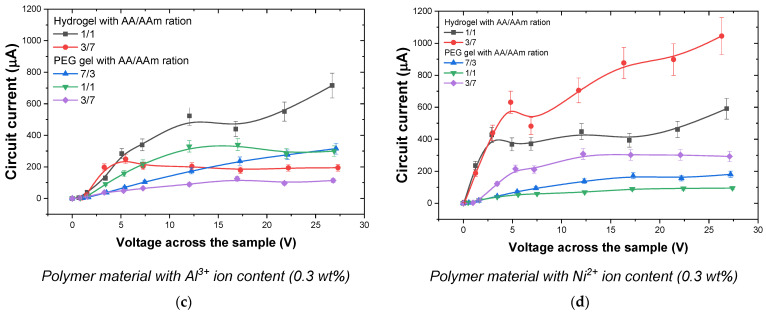
The I-V characteristics of hydrogels and PEG-gels are based on acrylic acid (AA) and acrylamide (AAm) with different mass ratios of monomers and modifiers (0.3 wt.% of metal ions): (**a**) unmodified gels; (**b**) gels modified Fe^3+^; (**c**) gels modified Al^3+^; (**d**) gels modified Ni^2+^.

Figure 7 shows the volt-ampere characteristics of pure hydrogel samples and PEG gels based on acrylic acid (AA) and acrylamide (AAm) with different mass ratios of monomers.

It was found that the electrical conductivity of hydrogels depends significantly on the AA/AAm ratio. The sample with a predominance of acrylamide (AA/AAm = 3/7) is characterized by the highest conductivity. At a voltage of 26.3 V, the current in the circuit reaches 1009.2 μA, which is more than 2.7 times higher than the maximum current value for the AA/AAm = 1/1 composition (374.3 μA at 27.0 V). The I-V characteristics of both samples are nonlinear: after an initial section with low conductivity, a sharp increase in current is observed at voltages above 1.5–3.0 V, which is typical of systems with mobile counterions (K^+^) in the polymer matrix.

The introduction of PEG, a non-ionic polymer, into the system predictably leads to a decrease in electrical conductivity in all the studied compositions. The most pronounced suppression of ion transport is observed for the PEG gel sample with an equal monomer ratio (AA/AAm = 1/1): the maximum current does not exceed 26.6 μA even at a voltage of 27.3 V, and the I-V characteristics are close to linear (ohmic), which is typical of materials with high resistivity. For the composition with a predominance of acrylic acid (AA/AA = 7/3), the highest absolute conductivity was recorded among the PEG-containing samples: the maximum current reaches 165.1 μA at 27.5 V. The PEG gel with a predominance of acrylamide (AA/AAm = 3/7) demonstrates a more stable and predictable current–voltage characteristic. The maximum current for this sample is 95.4 μA at 27.3 V, which is approximately an order of magnitude lower than that of a similar unmodified hydrogel.

The introduction of FeCl_3_ salt into hydrogels leads to a significant increase in conductivity compared to pure samples. For the 1/1 composition, the maximum current increases from 374 μA (pure) to 394 μA (modified) at 26.8 V. The I-V characteristics become more linear, which is characteristic of ohmic conductivity. This increase in conductivity is due to the introduction of additional Fe^3+^ ions, which increase the concentration of mobile charge carriers. In the presence of PEG, modification with FeCl_3_ also increases the conductivity of PEG gels relative to pure PEG, but the absolute values remain an order of magnitude lower than in hydrogels.

The introduction of AlCl_3_ salt into the gels has the most pronounced effect on the electrical conductivity of the hydrogels. For the 1/1 composition, the maximum current reaches 715.6 μA at 26.7 V, which is almost twice that of the pure hydrogel (374 μA). For the 3/7 composition, the maximum current is 249.5 μA at 5.48 V. The high conductivity is explained by the high charge of the Al^3+^ ion, which provides the highest concentration of mobile counterions and promotes additional dissociation of carboxyl groups. In PEG gels, modification with AlCl_3_ leads to the highest conductivity values among the studied ion modifiers. For the 7/3 composition, the maximum current is 315.6 μA at 27.1 V, which significantly exceeds the values of the pure PEG gel (211 μA) and PEG gels modified with FeCl_3_ salt. For the 3/7 composition, the maximum current reaches 114.6 μA.

The introduction of NiCl_2_ salt ensures high conductivity of hydrogels, comparable to AlCl_3_. For the 1/1 composition, the maximum current reaches 590.8 μA at 26 V, for the 3/7 composition—1044.9 μA at 26 V. A distinctive feature is a stable increase in current over the entire voltage range without pronounced plateaus or drops, which indicates stable ionic conductivity. In PEG gels, the introduction of NiCl_2_ salt demonstrates a moderate increase in conductivity relative to pure samples; however, the values remain low: the maximum current for the 7/3 composition is 180.7 μA at 27 V, for the 1/1 composition—94.5 μA (at 27 V). The highest values among PEG gels with NiCl_2_ were recorded for the 3/7 composition (308.3 μA at 12 V). For all PEG gel compositions, nonlinearity of the current–voltage characteristics with saturation regions is observed, which limits the practical application of these materials as conducting media.

### 2.5. Hysteretic I-V Characteristics of P(AA-Co-AAm) Hydrogels and PEG-Based Organogels

To evaluate the hysteretic behavior, hydrogel and PEG gel samples with a monomer ratio of AA/AAm = 1/1, modified with NiCl_2_, AlCl_3_, and FeCl_3_ salts, were studied. Measurements were conducted with cyclic changes in the input voltage, recording the voltage across the sample ($U_{mat}$) and calculating the current in the circuit ($I_{circuit}$). The data obtained was used to analyze the shape of the current–voltage characteristics (the I-V characteristics) and the presence of hysteresis.

**Figure 8:**
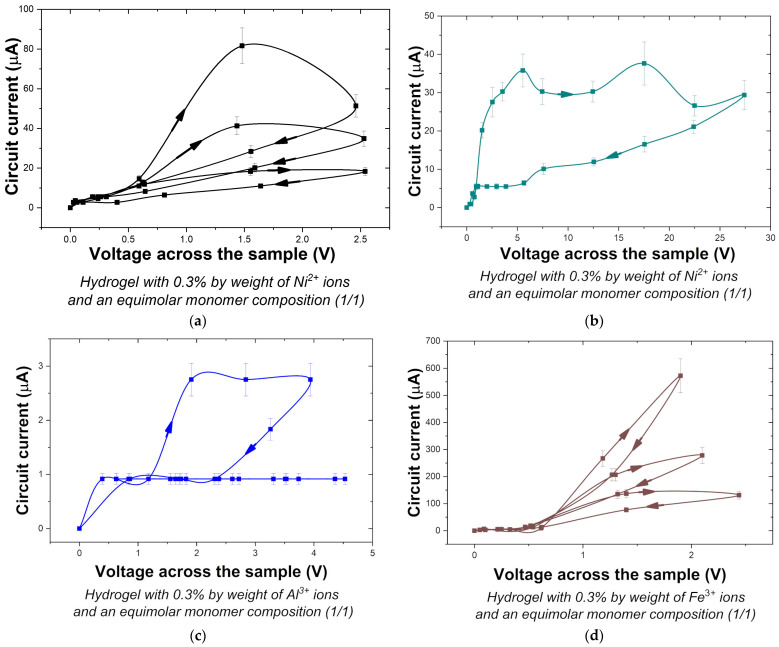
The dependence of the current (I) on the voltage (U) on the sample during cyclic scanning (3 forward and reverse cycles) for AA-AAm hydrogels with 1/1 monomer ratios with a 0.3 wt% content of (**a**,**b**) ${Ni}^{2+}$, (**c**) ${Al}^{3+}$, (**d**) ${Fe}^{3+}$. The arrows indicate the direction of the cycle. Conditions: (**a**,**c**,**d**) Voltage range (0 to 5 V), room temperature (25 °C); (**b**) Voltage range (0 to 30 V and 1 cycle), room temperature (25 °C). The observed pinched hysteresis loops may be influenced by ion migration, electrode polarization, and other electrochemical effects; therefore, the behavior is described as hysteretic I-V characteristic rather than definitive memristive behavior.

Figure 8a,b shows the I-V curves of the hydrogel modified with Ni^2+^ ions, studied in low and high voltage ranges. A characteristic hysteresis loop is observed during cycling in the 0–5 V range. In the first cycle, the current increases nonlinearly during the forward stroke (increasing voltage), while during the reverse stroke (decreasing voltage), the current values at the same voltage are lower than during the forward stroke. The resistance changes from tens of kΩ during the first cycle to hundreds of kΩ during the final cycle ($R_{cp1}=54.7\;k\Omega \to R_{cp1}=78.8\;k\Omega \to R_{cp1}=148.9\;k\Omega$). In successive cycles, a gradual increase in resistance is observed: if in the first cycle at $U_{\mathrm{init}}=5$ V, the resistance was ~47.9 kΩ, then in the second cycle it increases to ~72.6 kΩ, and in the third—to ~81.7 kΩ. This behavior may be associated with thermal effects and moisture loss under the action of direct current, which leads to conductivity degradation, however, hysteresis is maintained in all cycles. When the range is extended to 30 V (Figure 7b), pinched hysteresis loops also manifest themselves. The I-V characteristic has a pronounced hysteresis loop, and the sample resistance non-monotonically depends on the applied voltage: at low values ($U_{\mathrm{init}}<5$ V), it is high (hundreds of kΩ—units of MΩ), then decreases, reaching a minimum in the range of 20–25 V, and then increases again.

The aluminum chloride-modified hydrogel also exhibits hysteresis in the first cycle (0–5 V): at $U_{\mathrm{init}}=5$ V, the current is 2.75 μA, and the resistance is ~1.43 MΩ. On the reverse cycle, at the same voltage, the current decreases and the resistance increases. However, in the second and subsequent cycles, the current barely exceeds 0.92 μA (the minimum value limited by the measurement sensitivity), and the voltage across the sample becomes close to the applied voltage. This indicates a sharp increase in resistance (over 10 MΩ) and a loss of hysteresis. Thus, AlCl_3_ is characterized by rapid irreversible passivation of the sample, likely due to the rapid evaporation of water from the hydrogel and the formation of a dense insulating layer. For the FeCl_3_-modified hydrogel, the hysteresis loop is clearly visible in the 0–5 V range (Figure 8d). In the first cycle at 5 V, the resistance is ~3.3 kΩ, which is significantly lower than that of NiCl2. In subsequent cycles, the resistance increases (to ~7.6 kΩ in the second cycle and to ~18.6 kΩ in the third). The hysteresis is maintained, but the loop shape changes. Compared to NiCl_2_, FeCl_3_ provides higher initial conductivity, but also exhibits parameter drift with repeated cycling.

**Figure 9:**
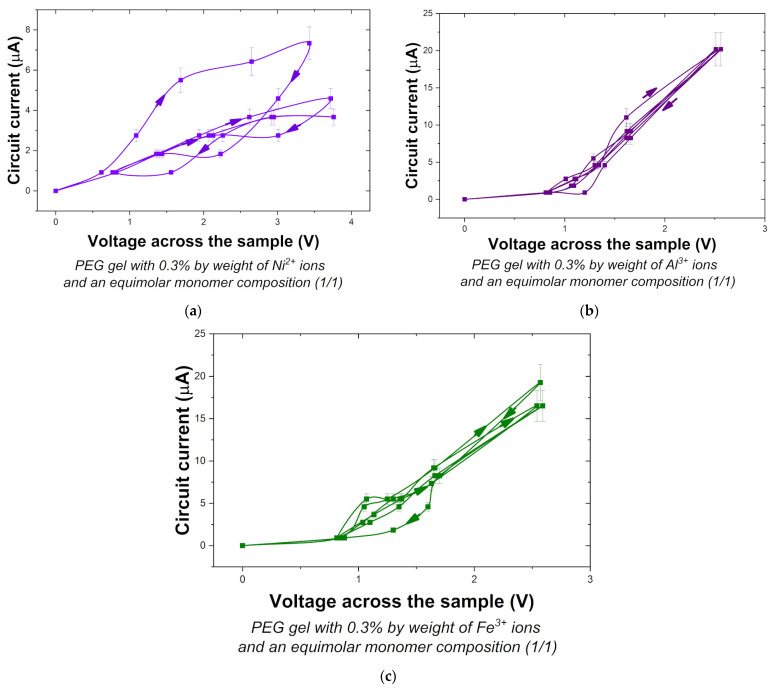
The dependence of the current (I) on the voltage (U) on the sample during cyclic scanning (3 forward and reverse cycles) for AA-AAm PEG-gels with 1/1 monomer ratios with a 0.3 wt% content of (**a**) ${Ni}^{2+}$, (**b**) ${Al}^{3+}$, (**c**) ${Fe}^{3+}$. The arrows indicate the direction of the cycle. Voltage range (0 to 5 V), room temperature (25 °C). The observed pinched hysteresis loops may be influenced by ion migration, electrode polarization, and other electrochemical effects; therefore, the behavior is described as hysteretic I-V characteristic rather than definitive memristive behavior.

A weak hysteresis loop is observed in I-V characteristics for the PEG gel modified with NiCl_2_ in the 0–5 V range (Figure 9a). The absolute current values are significantly lower than in the hydrogel: at 5 V, the current is 7.34 μA (resistance ~467 kΩ) in the first cycle. In the second cycle, the resistance increases to ~1.02 MΩ, and in the third, it decreases slightly (~810 kΩ), remaining above the initial value. Hysteresis is reproduced in all cycles, but its area is significantly smaller than in the hydrogel. Compared to hydrogel, the PEG gel has a higher overall resistance and less pronounced drift of its parameters, which is likely due to its greater structural stability.

Unlike the hydrogel, where AlCl_3_ caused rapid irreversible passivation, the PEG gel sample with AlCl_3_ does not cause locking (Figure 9b). In the first cycle at 5 V, the resistance is ~124 kΩ, and on the reverse cycle, ~181 kΩ. In subsequent cycles, the parameters stabilize: at 5 V, the resistance remains within 125–127 kΩ. However, hysteresis in the I-V characteristics is virtually absent—the curves for the forward and reverse cycles are close to linear, and the loop is indistinguishable due to measurement error. Thus, replacing water as a solvent with PEG-400 prevents irreversible locking but does not provide a pronounced hysteretic response.

A PEG gel sample modified with FeCl_3_ salt exhibits very weak hysteresis (Figure 9c). The resistance in the first cycle at 5 V is ~157 kΩ. In subsequent cycles, resistance values fluctuate between 133 and 154 kΩ. The hysteresis loop has a small area and is not always reliably reproduced; in some cycles, it may be absent. The I-V characteristic approaches an ohmic one, indicating the dominance of linear conductivity.

For a quantitative comparison of hysteresis loops, their areas were calculated (Table 4, Table 5 and Table 6).

In hydrogel, the largest average area is given by FeCl_3_ (51.7 µA·V), followed by ${NiCl}_{2}$ (30.5 µA·V); AlCl_3_ shows only 1.5 µA·V. In the PEG gel, measurable hysteresis is preserved only for NiCl_2_ (3.06 µA·V), which is almost an order of magnitude lower than in the hydrogel. There are no reproducible positive loops in the PEG matrix for AlCl_3_ and FeCl_3_.

The trends of area change from cycle to cycle (Table 5) differ: ${NiCl}_{2}$ and ${AlCl}_{3}$ hydrogels have negative slopes (−20.4 and −3.0 µA·V/cycle), indicating rapid degradation or passivation. On the contrary, FeCl_3_ in hydrogel demonstrates a unique positive trend (+4.71 µA·V/cycle) with extremely low variability (CV = 0.05), which indicates self-amplification of hysteresis (probably due to Fe^3+^/Fe^2+^ redox processes). In the PEG gel, all trends are negative or undetectable.

The area changes from the first to the last cycle (ΔS, Table 6) confirms these conclusions: for NiCl_2_ hydrogel ΔS = −40.7 µA·V (rapid degradation), for AlCl_3_ ΔS = −3.0 µA·V (disappearance of hysteresis after the first cycle), for ${FeCl}_{3}$ ΔS = +4.71 µA·V (growth). In PEG gel, NiCl_2_ yields ΔS = −5.6 µA·V (slow degradation), while AlCl_3_ and FeCl_3_ do not provide reproducible loops (ΔS ≈ 0).

### 2.6. Discussion

As is known [33,34], the ratio of acrylic acid (AA) to acrylamide (AA) is a key factor determining the architecture of the polymer network, crosslinking density, hydrophilicity, and mechanical properties of the synthesized hydrogels. Changes in the hydrogel network structure shown in Table 2 can be explained by a number of factors related to the reactivity of the comonomers and the characteristics of the formation of the network structure during radical copolymerization [35]. Analysis of unmodified hydrogels revealed a clear relationship between the copolymer composition and the characteristics of the polymer network: with an increase in the proportion of acrylamide, the storage modulus increases by 3.4 times, the crosslinking density increases by 3.5 times, and the average mesh size decreases by 34%. This behavior can be explained by the fact that acrylamide has a higher chain growth rate constant, and carboxylate ions create electrostatic repulsion, loosening the network. Moreover, with an excess of AA, the crosslinking agent (MBA) is realized more effectively.

The comonomer ratio determines not only the properties of the original polymer matrix but also the nature of the interaction of the introduced metal ions with the functional groups of the copolymer [30]. The results show that the effectiveness of reinforcement or softening with the introduction of metal salts depends significantly on the availability of carboxyl groups, which act as the main coordination centers.

To visualize the dependence of modification efficiency on the polymer matrix composition, the relative change in storage modulus ($\Delta G_{e}$, %) was calculated for each modifier relative to the pure sample at the corresponding monomer ratio. The obtained values are summarized in Table 7.

The presented data allows us to identify several key patterns confirming the decisive role of the monomer ratio in the efficiency of ionic modification.

For samples modified with Ni^2+^ and Al^3+^ ions, a clear dependence of the sign and magnitude of the effect on the monomer ratio is observed. At an acrylic acid content of 50% or higher, modification leads to an increase in the storage modulus. The maximum reinforcing effect was recorded for the hydrogel (1/1) modified with AlCl_3_ salt, where $\Delta G_{e}$ reaches +53.55%. For the hydrogel modified with NiCl_2_ in the same system, the increase in the storage modulus is +14.08%. With a decrease in the AA proportion to 30%, the effect becomes negative: the storage modulus decreases by 22.57% for AlCl_3_ and by 53.56% for NiCl_2_. This may indicate a change in the mechanism: with a lack of carboxyl groups, the coordination interaction can no longer compensate for the structural disturbances introduced by the additive.

Modification of hydrogels with iron salts in all studied compositions leads to a decrease in the storage modulus, but the degree of severity of the destructive effect depends significantly on both the nature of the anion and the ratio of monomers in the polymer matrix.

In all studied formulations, the addition of FeCl_3_ exhibited a negative effect, but its magnitude depended significantly on the monomer ratio. The smallest decrease in modulus was recorded for the equimolar system (−31.78%), while the destructive effect was more pronounced with excess acrylic acid (−37.81%) and acrylamide (−68.61%).

However, attributing the softening solely to radical inhibition by Cl^−^ anions is an oversimplification. In ${FeCl}_{3}$-modified systems, ${Fe}^{3+}$ ions can simultaneously act as physical crosslinkers via coordination with carboxylate groups ($-{COO}^{-}\cdots {Fe}^{3+}$) [36,37,38] and as disruptors due to the high Lewis acidity of ${Fe}^{3+}$, which may catalyze local hydrolysis of amide groups or cause chain scission [39,40]. The net mechanical effect depends on the balance between these competing processes. In contrast to ${Al}^{3+}$, which forms stable and kinetically inert coordination bonds, ${Fe}^{3+}$ complexes are more labile and can undergo ligand exchange with ${Cl}^{-}$, water, or PEG [41,42]. This lability prevents the formation of a permanent reinforcing network and instead leads to a dynamic, easily disrupted structure [43]. Furthermore, the presence of chloride anions can accelerate corrosion-type reactions in the polymer matrix, especially under the acidic conditions generated by incomplete neutralization of acrylic acid [44]. A clear distinction between anion-induced inhibition of polymerization and post-synthetic coordination-induced network weakening remains an open question. Future studies combining X-ray photoelectron spectroscopy (XPS) and rheology under controlled conditions are needed to decouple these mechanisms.

Fe(SO_4_)_2_(NH_4_)_2_ as a modifier demonstrates more pronounced softening compared to FeCl_3_ in all systems.

FeC_2_O_4_ salt exhibits the most destructive effect of all the studied compositions. The relative reduction in storage modules ranges from −87.95% to −89.43%, regardless of the monomer ratio. Such significant and universal softening confirms the decisive role of the anionic environment: oxalate ions, with their pronounced reducing properties, are capable of effectively inhibiting radical polymerization, significantly reducing the molecular weight of the resulting polymer and the degree of crosslinking, regardless of the composition of the reaction mixture.

Analyzing the effects of three iron salts, we can conclude that the destructive effect is determined not so much by the nature of the cation (iron is used in all cases) as by the anionic environment. In the series $FeCl_{3}$ → $FeSO_{4}(NH_{4}{)}_{2}SO_{4}$ → ${FeC}_{2}O_{4}$, a consistent increase in the degree of weakening of the polymer matrix is observed, which correlates with the increase in the reducing power of the anions. $FeCl_{3}$ is the least aggressive modifier of the iron salts studied, while $FeC_{2}O_{4}$ almost completely suppresses the formation of a regular polymer network, regardless of the composition of the reaction mixture.

The multidirectional effects of metal salts on the structural parameters of gels differ significantly for hydrogels and PEG gels. A comparison of the data allows us to identify two characteristic types of modifier effects depending on the presence of PEG.

It is important to note that the transition from an aqueous medium to a system with a high content of PEG-400 fundamentally changes the conditions for the formation of a polymer mesh. Polyethylene glycol, being a highly viscous solvent with a pronounced ability to form hydrogen bonds, performs several functions:

Firstly, PEG-400, being embedded in a polymer matrix, increases the mobility of polymer chains and reduces the density of physical crosslinking due to competition for hydrogen bonds [45].

Secondly, the high viscosity of the medium slows down the diffusion of monomers, initiator, and growing radicals, which can lead to a decrease in the degree of polymerization and a change in the topology of the mesh (the degree of branching, the presence of gels/moons) [46,47].

Thirdly, PEG-400 does not participate in covalent crosslinking, but it can form physical bonds with the polymer matrix, creating additional noncovalent nodes that are reversibly destroyed by mechanical stress and restored after removal of the load.

Moreover, the high content of PEG-400 affects the solubility of metal salts and their ability to complex with carboxyl groups of the polymer.

For example, in the absence of PEG, the addition of AlCl_3_ predictably increases crosslinking density in all formulations, but this effect is moderate (Table 2). In the presence of PEG, the addition of AlCl_3_ results in the most pronounced strengthening of all modifiers. This indicates a synergistic effect between PEG and Al^3+^ ions: PEG-400 likely promotes more efficient coordination of aluminum ions with carboxylate groups through the formation of PEG–cation complexes, which enhances ionic crosslinking [48,49].

In both series, the introduction of FeCl_3_ into the gel results in a decrease in crosslinking density relative to the pure gel; however, in the presence of PEG, this effect is more pronounced. This may be due to competition between PEG and carboxylate groups for coordination with Fe^3+^ ions [50]. PEG-400, with its high donor capacity for iron cations, can form stable complexes, removing ions from the process of intermolecular crosslink formation.

The most striking difference is observed for NiCl_2_. In the absence of PEG, nickel ions act as structure-forming agents (increasing nₑ). In the presence of PEG, on the contrary, NiCl_2_ in all formulations either slightly reduces or maintains the crosslinking density at the level of the pure gel. This may be since Ni^2+^, being a medium-strength ion, effectively interacts with carboxylate groups in the absence of PEG, while in the presence of PEG, it preferentially coordinates with the ether oxygens of the ethylene glycol chains, weakening its role as a crosslinking agent for the polymer matrix.

Among the studied modifiers, the double iron (II) salt, Mohr’s salt (Fe(NH_4_)_2_(SO_4_)_2_), exhibits a pronounced tendency toward phase separation in the polymer matrix, accompanied by the formation of poorly soluble iron-PEG complexes on the surface and in the bulk of the samples. Moreover, a comparison of the structural parameters in systems with and without PEG reveals fundamentally different effects of this modifier on the network structure. In the absence of PEG, the addition of Mohr’s salt leads to a sharp increase in the distance between crosslinks, especially for the acrylamide-dominated composition (3/7), where ξ nearly doubles—from 3.78 to 7.39 nm. This indicates a significant loosening of the network structure. In the presence of PEG, the increase in ξ is significantly weaker (from 12 to 22%), indicating a fundamentally different mechanism of interaction between the modifier and the polymer matrix.

Based on the data on self-healing presented in Table 3, it is possible to formulate some key patterns that determine the efficiency of self-healing of composite polymer materials.

The efficiency of the process is determined by three interrelated factors: the monomer ratio (AA/AAm), the nature of the metal modifier, and the type of solvent. The monomer ratio determines the balance between carboxyl (ionogenic) and amide groups, determining the accessibility of coordination sites and the mobility of the chains. The nature of the metal modifier determines the strength and reversibility of the coordination bonds: reversible sites facilitate recovery, while irreversible ones hinder it [43]. PEG-400, which replaces water in the hydrogel, acts as a plasticizer, increases free volume, and participates in the formation of a dynamic hydrogen bond network [51].

In most cases, high self-healing efficiency (70–100%) correlates with a decrease in crosslinking density (nₑ) and an increase in the network cell size (ξ) relative to the pure sample. The loose structure ensures the necessary mobility of polymer chains for their recombination after degradation.

In some cases, high efficiency is achieved even with increasing crosslinking density, as occurs in the PEG gel modified with Al^3+^: nₑ increases from 14.28 to 27.07 mol/m^3^, ξ decreases to 3.94 nm, but the efficiency remains 79.98%. This indicates that the key factor is not the absolute density of the network, but the reversibility of ionic coordination bonds, which can be rearranged under load. In Table 8, a ranking is provided of the modifiers by versatility for enhancing self-healing efficiency.

Thus, for maximum self-healing in hydrogels and PEG gels, compositions with an equimolar monomer ratio modified with FeCl_3_ or Fe(NH_4_)_2_(SO_4_)_2_ are optimal. The most universal modifier for both types of materials is Fe(NH_4_)_2_(SO_4_)_2_, which ensures consistently high self-healing rates (51–72% in hydrogels, 48–100% in PEG gels) due to an optimal balance between network loosening and reversibility of coordination bonds. It can also be assumed that achieving high self-healing efficiency requires either sufficient chain mobility by reducing crosslinking density or high reversibility of ionic nodes capable of dynamic rearrangement [52].

The structure of the polymer network can also explain the electrical properties of the gels. The highest conductivity of the acrylamide-dominated composition (1009.2 μA at 26.3 V) and the nonlinear I–V characteristics can be explained by the fact that this composition has a higher crosslinking density and a smaller cell size, resulting in a more uniform network that facilitates efficient ion migration [53,54]. The 1/1 composition, despite its lower formal crosslinking density, has additional physical nodes due to electrostatic interactions, which reduces charge carrier mobility.

The introduction of PEG (a non-ionic polymer) naturally reduces the conductivity by an order of magnitude in all compositions, resulting in the worst conductivity and a current–voltage characteristic close to linear (ohmic) in the 1/1 PEG gel composition.

Comparing the efficiency of ionic modifiers in hydrogels and PEG gels, we can write the conductivity series Al^3+^ ≈ Ni^2+^ > Fe^3+^ (for hydrogels) and Al^3+^ > Ni^2+^ > Fe^3+^ (for PEG gels). In hydrogels, NiCl_2_ and AlCl_3_ salts provide the highest conductivity (up to ~1000 μA), while FeCl_3_ is less effective, in some cases reducing conductivity. For PEG gels, AlCl_3_ proves to be the most effective (315.6 μA); the I–V characteristics do not reach a plateau, and NiCl_2_ and FeCl_3_ salts show a moderate increase with saturation.

The reduction in ion mobility in the PEG gel compared to the hydrogel results in suppression of hysteresis. However, the degree of hysteresis behavior depends significantly on the nature of the cation. The most noticeable hysteresis in PEG gels is observed for modification with the Ni^2+^ salt, whereas for modification with the Al^3+^ and Fe^3+^ salts, it is virtually absent or extremely weak. This may be due to several factors. First, PEG can form coordination complexes with metal cations via the oxygen atoms in the ethylene oxide units. The binding energy of Ni^2+^ to the ether oxygens of PEG is lower than that of Al^3+^ and Fe^3+^, which are harder Lewis acids and form more stable complexes [52,53,55]. As a result, nickel ions retain relatively high mobility in the matrix, allowing some migration under the influence of the electric field and, consequently, weak hysteresis. Secondly, Ni^2+^ exhibits a higher ligand exchange rate compared to Al^3+^ and Fe^3+^. This means that the Ni^2+^ bonds with the polymer ligands are more easily reversibly broken and reformed upon changing the applied field. Such dynamism could contribute to the formation of hysteresis; however, in a viscous PEG medium, this effect can be greatly suppressed. Furthermore, Al^3+^, possessing a high charge and small radius, forms extremely stable complexes with the ether oxygens of PEG, which almost completely suppresses its mobility. Fe^3+^ also exhibits high complexing capacity (albeit somewhat less than Al^3+^), which explains the absence of pronounced hysteresis in this case. Thus, only Ni^2+^ is the most promising modifier for PEG-containing systems, since it maintains weak but reproducible hysteresis, provides better stability of parameters and balances between mobility and reversibility of bonds.

It should be emphasized that the hysteretic I-V loops observed in Ni^2+^-modified gels (Figure 8 and Figure 9) are not unequivocal proof of true memristive behavior. Alternative mechanisms such as ion migration, redox reactions (e.g., Fe^3+^/Fe^2+^ or Ni^2+^/Ni^0^), electrode polarization, water electrolysis, and moisture loss under applied voltage can all contribute to the observed nonlinearity and hysteresis. This is particularly relevant for measurements at voltages above 5 V (Figure 9b). Therefore, we refrain from claiming genuine memristive behavior. Instead, we describe the results as pinched hysteresis loops that are consistent with reports of ionic memristive systems but require further validation through controlled experiments (e.g., impedance spectroscopy, inert atmosphere, inert electrodes, and variable sweep rates).

The presented results demonstrate a complex dependence of the hysteretic I-V behavior on the nature of the polymer matrix, the type of solvent, and the type of cation. Future work will focus on a more detailed analysis of the cyclic I–V characteristics (at least 10 cycles) to assess the long-term stability and reproducibility of the observed hysteretic behavior.

## 3. Conclusions

This study demonstrates for the first time the possibility of targeted multiparametric regulation of the functional properties of composite polymer materials through the synergistic combination of three independent factors: the architecture of the polymer network (the AA/AAm ratio), the nature of the ionic modifier, and the type of solvent (water/PEG-400). Increasing the proportion of acrylamide (from 7/3 to 3/7) ensures a monotonic increase in mechanical rigidity (the storage modulus increases by a factor of 3.4: 22.0 → 76.2 kPa) due to an increase in the crosslinking density and the formation of a more homogeneous network. Replacing 70% of water with PEG reduces the elastic modulus (plasticizing effect) but opens access to unique synergistic effects. The PEG matrix provides record self-healing efficiency (~100% for Fe^3+^ at 1/1) due to a dynamic network of hydrogen bonds. Replacing water as a solvent with PEG-400 leads to the suppression of irreversible passivation (for PEG gels modified with Al^3+^ salts) but reduces ionic mobility and conductivity by an order of magnitude.

The modifiers used in this study, Al^3+^ and Ni^2+^ salts, exhibit a reinforcing effect in systems with high acrylic acid contents (ΔGₑ up to +53.5%), forming metal-carboxylate coordination nodes. Iron salts cause softening (up to −90% for FeC_2_O_4_) due to the inhibition of polymerization by redox processes. The samples containing Ni^2+^ exhibit reproducible closed hysteresis loops. However, it remains unclear whether this hysteresis reflects genuine memristive switching or arises from electrochemical artifacts such as ion migration or electrode polarization. Controlled experiments under an inert atmosphere and using inert electrodes are required to elucidate the underlying mechanism. Quantitative analysis of the areas of hysteresis loops shows that the average area of NICL_2_ hydrogels is 30.5 µA·V, while in PEG gels it decreases to 3.1 µA·V. FECL_3_ hydrogels show a unique positive tendency to increase the area during cycling (+4.7 µA·V per cycle) with very low variability, which indicates self-amplification due to redox processes, while alcl_3_ causes rapid passivation in hydrogels (hysteresis disappears after the first cycle), but behaves ohmically in PEG-gels. Future work should include control experiments under an inert atmosphere, with inert electrodes (e.g., platinum), and at varying sweep rates to distinguish true memristive behavior from artifactual electrochemical effects.

Thus, the combination of varying copolymer composition, the nature of the metal modifier, and the type of solvent allows for wide-ranging control of the mechanical, electrical, and adaptive properties of polymer gels, opening prospects for creating next-generation materials for soft robotics, bioelectronics, neuromorphic computing, and self-healing devices.

## 4. Materials and Methods

### 4.1. Materials

A series of composite polymer hydrogels, modified with various metal ions, was synthesized via free-radical crosslinking copolymerization in an aqueous solution. The synthesis protocol was systematically adapted to produce materials with three distinct monomer ratios and varying concentrations of metal ions to investigate their effect on the final properties.

The primary monomers used were acrylamide (AAm, CJSC Vecton, Saint Petersburg, Russia) and 80% partially neutralized acrylic acid (AA, CJSC Vecton, Saint Petersburg, Russia). Three different AA/AAm weight ratios were employed: 7/3, 1/1, and 3/7. To facilitate a comparative analysis of the polymer matrix’s behavior, the metal ion concentration was fixed at 0.3 wt%, as this value was identified as the most effective for comparison.

N,N’-methylenebisacrylamide (MBAa, CJSC Vecton, Saint Petersburg, Russia) served as the crosslinking agent, added at a concentration of 0.2 wt% relative to the total monomer mass. The polymerization reaction was initiated using a redox system consisting of ammonium peroxydisulfate (PSA, JSC Lenreactiv, Saint Petersburg, Russia) and the accelerator N,N,N’,N’-tetramethylethylenediamine (TEMED, SIGMA-ALDRICH, St. Louis, MO, USA).

In addition to the aqueous synthesis, a gel was prepared using the same AA/AAm ratios and crosslinker concentrations, but with distilled water replaced by polyethylene glycol 400 (PEG-400, OOO GK RusChem, Saint Petersburg, Russia). In this case, the metal salt concentration was also maintained at 0.3 wt% relative to the total monomer mass.

The modification was achieved by incorporating one of several different metal salts. The following types of salts were used: (1) iron (II) ammonium sulfate; (2) iron (III) chloride; (3) nickel (II) chloride; and (4) aluminum (III) chloride.

### 4.2. Synthesis of P(AA-Co-AAm)/Me^n+^ Salts Hydrogels

The general synthesis procedure for all hydrogels followed a sequential, multi-step process. The hydrogels were polymerized by taking the mole ratio of AAm and AAc as 70:30, 50:50 and 30:70, which are designated 7/3, 1/1 and 3/7, respectively, and are shown in Table 9.

A detailed description of the synthesis for a sample with a 70/30 monomer ratio, a target total monomer weight of 16.72 g, and a defined crosslinking degree is provided below. The synthesis for other monomer ratios and salt concentrations was performed analogously, with the quantities of monomers adjusted accordingly while maintaining the total monomer weight.

First, 16.20 g of distilled water was heated to 40 °C in a glass beaker under constant stirring (300 rpm). This temperature was consistently maintained throughout the entire synthesis process (Stage 1). A precisely calculated amount of the chosen metal salt (iron salts, ${NiCl}_{2}$, or ${AlCl}_{3}$) was then added to the heated water and allowed to dissolve completely for 2 min.

Next, the monomer mixture was introduced. For the 7/3 ratio, this consisted of 13.29 g of the partially neutralized acrylic acid solution and 3.43 g of acrylamide. The solution was stirred for 4 min to ensure a homogeneous mixture. Subsequently, 0.05 g of the crosslinking agent, N,N’-methylenebisacrylamide (MBA), was added and stirred for another 2 min. Following this, 2.86 mL of an 8% aqueous solution of TEMED was added to the reaction vessel to catalyze the polymerization.

The polymerization reaction was initiated by the final addition of 2.86 mL of an 8% aqueous ammonium persulfate (PSA) solution. The entire reaction mixture was vigorously stirred for 10 s to ensure uniform distribution of the initiator and was then immediately poured into an airtight polypropylene container. The polymerization was allowed to proceed at room temperature without further stirring.

The total mass of the reaction mixture was 38.68 g. Upon completion of the synthesis, all hydrogel samples were stored in a refrigerator at 6 °C.

### 4.3. Synthesis of P(AA-Co-AAm)/Me^n+^ Salts PEG-Gels

For comparative analysis and improvement of the self-healing ability of polymer hydrogels, a modification of the reaction medium was applied, in which most of the water (70 wt.%) was replaced by polyethylene glycol with a molecular weight of 400 Da (PEG-400).

The synthesis of hydrogels in PEG-400 medium was carried out according to a procedure similar to that described in Section 4.2, while maintaining all concentration parameters (ratio of monomers, content of crosslinking agent, initiator, and concentration of modifiers). However, due to the partial insolubility of some salts in the highly viscous PEG medium (in particular, the Mohr salts Fe_2_(NH_4_)_2_(SO_4_)_2_∙6H_2_O), the modifiers were preliminarily dissolved in 2 mL of distilled water before being added to the reaction mixture. This approach ensured a homogeneous distribution of ionic additives in the system.

### 4.4. Dynamic Mechanical Analysis (DMA)

The viscoelastic properties of the synthesized hydrogels were investigated using a Physica MCR 502 rotational rheometer (Anton Paar, Graz, Austria) equipped with a parallel-plate measuring system (PP50, upper plate diameter of 49.95 mm), conforming to ISO 3219 standard [56]. All measurements were performed at a controlled temperature of 25.0 ± 0.2 °C maintained by a Peltier temperature control module (C-PTD200). Prior to analysis, the hydrogel samples were conditioned in a desiccator for 72 h to ensure a consistent hydration level and eliminate any residual stresses.

Amplitude sweep tests were conducted at a fixed angular frequency of ω = 10 Hz over a wide range of shear strains (γ = 0.001% to 500%) and *G*′, *G*″ values were recorded. Then, the values of the linear viscoelastic range (LVE-R) and the LVE-R limit were determined from the DMA curves.

Based on the dynamic mechanical analysis (DMA) data, key mesh parameters were calculated, including the mesh size (*ξ*) and the crosslinking density (*n_e_*). These parameters were determined using the following equations [57]:

(1) $$\xi ={\left(\frac{G_{e}\times N_{A}}{RT}\right)}^{-1/3}={\left(n_{e}\times N_{A}\right)}^{-1/3},$$

(2) $$n_{e}=\frac{G_{e}}{RT},$$

where $G_{e}$ is the equilibrium storage modulus in the LVE-R region (Pa), $N_{A}$ is Avogadro’s number (6.022 × 10^23^ mol^−1^), *R* is the universal gas constant (8.314 J·mol^−1^·K^−1^), and *T* is the absolute temperature (K). Equation (1) provides an estimate of the average distance between crosslinking points, while Equation (2) quantifies the concentration of elastically active chains within the polymer mesh.

### 4.5. FTIR Spectroscopy

Attenuated total reflectance (ATR)-FTIR was used to obtain deeper structural information and to study the water molecule subpopulation in the prepared AA-co-AAm/Me^n+^ hydrogels. Steady-state FTIR spectra were obtained by the ATR method using a Tensor 37 spectrometer (Bruker, Ettlingen, Germany). All experiments were carried out at room temperature, with a temperature of 25 °C controlled by an air conditioner. Steady-state FTIR spectra were recorded in the range of 4000–600 cm^−1^ with a resolution of 2 cm^−1^ and represented an average of 32 scans. The spectrum of a pure dry diamond ATR crystal in the ambient atmosphere was used as a background for the infrared measurements.

### 4.6. SEM-EDX

Attenuated total reflectance (ATR)-FTIR was used to obtain deeper structural information. The chemical composition of the hydrogels and PEG-gels was studied using energy dispersive X-ray spectroscopy (EDX) (Ultim MAX 100 with AZtec 5.0 software, Oxford Instruments, Oxford, UK) on a TESCAN scanning electron microscope (SEM) (TESCAN ORSAY HOLDING, a.s.; Brno-Kohoutovice, Czech Republic) with a spectrum analyzer attachment (Oxford Instruments plc, Abingdon, Oxford, UK). The analysis was carried out at the 20 keV energy range and 800,000 count limit. The measurements were carried out on samples dried at room temperature for 3 days. On each sample, the EDX spectra were measured at four points.

### 4.7. Thermal Analysis

A commercial DSC of the type DSC 204 F1 Phoenix from NETZSCH (Selb, Germany) was used for the measurements. Dry nitrogen gas with a flow rate of 50 mL min^−1^ was purged through the DSC cell. An aluminum hermetic pan was used for measurement. The sample mass was about 15–20 mg. The samples were pre-cooled at −80 °C by a scanning rate of 3 °C min^−1^ and kept for 5 min before the heating process of DSC experiments. DSC measurements were carried out under a constant heating rate of 3 °C min^−1^.

The proportion of freezing water from all water in the hydrogel according to DSC data can be determined by the formula:

(3) $$X_{f}=\frac{\Delta H_{m}}{\Delta H_{m}^{0}}\cdot \frac{\Delta H_{ev}^{0}}{\Delta H_{ev}}\times 100\%,$$

where $\Delta H_{m}$ and $\Delta H_{ev}\;$ are the enthalpies of melting and evaporation of water in the hydrogel normalized to the mass of the gel (J/g), $\Delta H_{m}^{0}$ and $\Delta H_{ev}^{0}$ are the enthalpies of melting and evaporation of pure distilled water (J/g).

Then the proportion of non-freezing bound water:

(4) $$X_{b}=\left(1-\frac{\Delta H_{m}}{\Delta H_{m}^{0}}\cdot \frac{\Delta H_{ev}^{0}}{\Delta H_{ev}}\right)\times 100\%,$$

The masses of freezing and non-freezing bound water per mass of dry hydrogel can be calculated using the formulas (5):

(5) $$\frac{m_{b}}{m_{d.h.}}=\frac{\frac{\Delta H_{ev}}{\Delta H_{ev}^{0}}-\frac{\Delta H_{m}}{\Delta H_{m}^{0}}}{1-\frac{\Delta H_{ev}}{\Delta H_{ev}^{0}}}\;and\;\frac{m_{f}}{m_{d.h.}}=\frac{\frac{\Delta H_{m}}{\Delta H_{m}^{0}}}{1-\frac{\Delta H_{ev}}{\Delta H_{ev}^{0}}}.$$

Thermogravimetry of AA-co-AAm hydrogels with different salts was performed using a TG 209 F1 Libra analyzer (NETZSCH, Germany). Approximately 20 mg of fresh hydrogel was weighed in a corundum crucible. The heating temperature increased from 25 to 200 °C at a heating rate of 5 °C/min. At this drying stage, all residual moisture evaporates from the analyzed hydrogels. Dynamic thermogravimetry of the hydrogels was performed at nitrogen flow rates of 50 mL/min.

### 4.8. Electrical Conductivity Properties

The electrical properties of the gels were studied using a four-wire (Kelvin) circuit to eliminate the influence of contact resistance. Aluminum adhesive tape (foil tape) was used to attach the wires to the polymer sample; the tape ensured electrical contact over an area of approximately 0.5 cm at each end of the sample.

A DC power supply (0–30 V) and a digital multimeter (UNI-T UT33B+) were employed. For the current–voltage (I–V) curves, the voltage was increased stepwise from 0 to 30 V. The step was 2 V in the low-voltage region (0–10 V) and 5 V in the high-voltage region (10–30 V). This step size was chosen to keep the manual measurement time practical. At each voltage step, the multimeter reading was allowed to stabilize for 3–5 s before recording the current.

For cyclic measurements, the voltage was swept from 0 to 5 V and back to 0 in 1 V increments, with the same stabilization delay of 3–5 s per step. All measurements were carried out at room temperature (25 °C) in ambient air.

It should be noted that at voltages above 5 V, electrochemical effects (e.g., water electrolysis, redox reactions at the copper electrodes, electrode polarization) may contribute to the measured current. Therefore, the high-voltage region is interpreted qualitatively, while the low-voltage region (0–5 V) is used for comparative analysis of the intrinsic ionic conductivity. The cyclic I–V curves (0 → 5 → 0 V) are intended to reveal hysteresis.

To quantify the hysteresis loops, their area was calculated. For each cyclic volt-ampere characteristic (0 → 5 → 0 V), the loop area S was determined by numerically integrating the difference between the forward and reverse voltage branches of the current:

$$S=\;\int_{U_{min}}^{U_{max}}(I_{forward}\left(U\right)-I_{reverse}(U))dU$$

where and are the currents registered when the voltage increases and decreases, respectively. At least three independently prepared samples were measured for each sample; the average loop area and standard deviation were calculated. The change of S from cycle to cycle was also analyzed to assess the stability and degradation of hysteresis.

At least three independently prepared samples were measured for each composition.

Measurements were performed in a series electrical circuit containing a DC voltage source (1–30 V), a current-limiting resistor (1090 Ω), the polymer sample under study, and an LED load. The current in the circuit was calculated according to Ohm’s law (6) based on the voltage drop across the reference resistor.

(6) $$I=\frac{Ures}{Rres}.$$

The specific electrical conductivity σ of the material was calculated using formula (7)

(7) $$\sigma =\frac{l_{mat}}{R_{mat}\times S_{mat}},$$

where $R_{mat}$ is the calculated resistance of the material, $l_{mat}\;$ is the distance between the electrodes, $S_{mat}$ is the cross-sectional area of the sample.

### 4.9. Self-Healing of the Material

To simulate the degradation of the material (for the purpose of subsequent study of recovery), the samples were subjected to controlled mechanical stress, leading to a violation of the integrity of the polymer mesh. The damage was caused by stretching the samples until they burst on a universal electromechanical testing machine.

To evaluate self-healing efficiency, two sets of independent samples were prepared for each composition. The first set (control, *n* = 3) was tested to determine the original fracture strain (ε_ori_). The second set (*n* = 3) was first cut, then the halves were carefully joined for the healing procedure described below. Thus, the comparison was carried out between different samples before and after healing, and not on the same sample.

The process of repairing damaged samples was carried out in an airtight desiccator at room temperature. To create an environment with high humidity, distilled water was placed at the bottom of the desiccator, which ensured constant saturation of the atmosphere with water vapor and relative humidity close to 100%. The samples were placed on a perforated partition above the water mirror, excluding direct contact with the liquid phase to ensure the sorption of moisture exclusively from the vapor phase. The exposure time of the samples in the desiccator was 3 days.

The tensile testing of the samples of the obtained material was carried out according to the requirements on an Instron 5943 (Norwood, MA, USA) testing machine. Before starting the test, the following input parameters were selected: the test speed is 20 mm/min; the thickness of the samples is 5 mm; the length of the samples is 50 mm; the width of the samples is 10 mm.

A quantitative assessment of the self-healing ability of the material was carried out in relation to the fracture strain (*ε*) of the recovered sample to the fracture strain of the source material. Self-healing coefficient (*E_self_*, *%*) was calculated using the formula (8):

(8) $$E_{self}=\frac{\varepsilon_{after\;SH}}{\varepsilon_{ori}}\times 100\%$$

This conservative criterion (recovery from deformation) was chosen because it directly reflects the ability of the polymer mesh to restore chain mobility and the original topology, while strength or degree of fracture often shows incomplete or erroneous recovery due to residual defects or increased stiffness after healing [58].

At least 3 measurements were performed for each type of sample; the results were averaged and presented as an average value with a standard deviation.

## Figures and Tables

**Table 1:** Water content of hydrogels and PEG gels and some thermal properties.

Monomer Ratio (AA:AAm)	Modifier	Water Content According to TGA Data, %		m_f_/m_d.h._, g/g		m_b_/m_d_._h_., g/g		T_g_, °C	
		Hydrogel	PEG-Gel	Hydrogel	PEG-Gel	Hydrogel	PEG-Gel	Hydrogel	PEG-Gel
7/3	Pure	69.2	23.7	0.483	-	1.276	0.210	−37.4	−65.1
	NiCl_2_	67.2	23.9	0.486	-	1.990	0.204	−32.4	−64.9
	AlCl_3_	70.5	25.7	0.386	-	1.321	0.263	−35.9	−66.9
	FeCl_3_	68.0	24.6	0.365	-	1.181	0.226	−35.1	−68.9
	Fe(NH_4_)_2_(SO_4_)_2_	71.0	24.8	0.558	-	1.154	-	−37.7	-
1/1	Pure	69.5	22.9	0.444	-	1.354	0.196	−34.5	−65.2
	NiCl_2_	69.8	22.7	0.454	-	1.364	0.205	−33.2	−64.2
	AlCl_3_	69.8	24.7	0.497	-	1.333	0.246	−32.4	−66.9
	FeCl_3_	70.0	23.7	0.489	-	1.141	0.247	−36.4	−66.4
	Fe(NH_4_)_2_(SO_4_)_2_	70.1	24.1	2.500	-	3.825	-	-	-
3/7	Pure	70.4	17.6	0.381	-	1.161	0.179	−23.3	−71.0
	NiCl_2_	71.8	20.2	0.449	-	1.233	0.194	−24.6	−71.0
	AlCl_3_	72.3	21.4	0.469	-	1.336	0.204	−27.6	<75
	FeCl_3_	70.9	23.9	0.551	-	1.580	0.247	−33.3	−72.9
	Fe(NH_4_)_2_(SO_4_)_2_	76.8	18.6	1.223	-	2.994	-	-	-

**Table 2:** Mechanical properties and crosslinking densities of the AA-co-AAm hydrogels and PEG-gels modified with 0.3 wt %. different salts.

Monomer Ratio (AAAAm)	Modifier	LVE-R Limit, %		Equilibrium Storage Modulus (Gₑ), kPa		Crosslink Density (*n_e_*), mol/m^3^		Average Mesh Size (ξ), nm	
		Hydrogel	PEG-Gel	Hydrogel	PEG-Gel	Hydrogel	PEG-Gel	Hydrogel	PEG-Gel
7/3	Pure	8.1 ± 0.23	3.6 ± 0.18	22.04 ± 0.27	15.87 ± 0.79	8.9 ± 0.25	6.4 ± 0.32	5.7 ± 0.28	6.4 ± 0.32
	NiCl_2_	3.3 ± 0.10	4.3 ± 0.22	24.56 ± 0.69	10.18 ± 0.51	9.9 ± 0.21	4.1 ± 0.20	5.2 ± 0.26	7.4 ± 0.37
	AlCl_3_	3.5 ± 0.11	1.4 ± 0.07	26.96 ± 0.48	33.17 ±1.66	14.7 ± 0.44	13.4 ± 0.67	4.8 ± 0.24	4.9 ± 0.25
	FeCl_3_	23.3 ± 1.16	5.2 ± 0.26	13.71 ± 1.10	21.52 ± 1.08	5.5 ± 0.28	8.7 ± 0.44	6.7 ± 0.34	5.8 ± 0.29
	Fe(NH_4_)_2_(SO_4_)_2_	90.0 ± 4.50	3.5 ± 0.18	9.60 ± 1.23	11.00 ± 0.55	3.9 ± 0.20	4.4 ± 0.22	7.5 ± 0.38	7.2 ± 0.36
	FeC_2_O_4_	480.0 ± 24.0	-	2.66 ± 1.35	-	1.1 ± 0.11	-	11.6 ± 0.58	-
1/1	Pure	19.87 ± 0.99	4.7 ± 0.24	30.02 ± 1.50	19.15 ± 0.96	12.1 ± 0.61	7.7 ± 0.39	5.1 ± 0.19	5.9 ± 0.30
	NiCl_2_	6.72 ± 0.34	6.9 ± 0.35	34.24 ± 1.71	7.91 ± 0.40	13.8 ± 0.69	3.2 ± 0.16	4.9 ± 0.25	8.0 ± 0.42
	AlCl_3_	2.49 ± 0.12	0.7 ± 0.04	46.09 ± 2.30	30.72 ± 1.54	18.6 ± 0.93	12.4 ± 0.62	4.5 ± 0.23	5.1 ± 0.26
	FeCl_3_	31.50 ± 1.58	15.8 ± 0.79	20.45 ± 1.02	17.40 ± 0.87	8.2 ± 0.41	7.0 ± 0.35	5.8 ± 0.29	6.2 ± 0.31
	Fe(NH_4_)_2_(SO_4_)_2_	37.90 ± 1.90	14.7 ± 0.74	14.89 ± 0.74	13.60 ± 0.68	6.0 ± 0.30	5.5 ± 0.28	6.5 ± 0.33	6.7 ± 0.34
	FeC_2_O_4_	250.0 ± 12.5	-	3.30 ± 0.33	-	1.3 ± 0.13	-	10.7 ± 0.54	-
3/7	Pure	3.6 ± 0,11	1.7 ± 0.05	76.23 ± 0.61	35.41 ± 1.06	30.7 ± 0.25	14.3 ± 0.43	3.8 ± 0.04	4.9 ± 0.15
	NiCl_2_	2.7 ± 0.08	1.2 ± 0.04	35.40 ± 0.52	14.61 ± 0.73	14.3 ± 0.21	5.9 ± 0.30	4.9 ± 0.03	6.5 ± 0.20
	AlCl_3_	1.8 ± 0.05	1.6 ± 0.05	59.02 ± 0.44	67.10 ± 2.01	23.8 ± 0.18	27.1 ± 0.81	4.1 ± 0.01	3.9 ± 0.12
	FeCl_3_	7.2 ± 0.22	4.9 ± 0.15	2.93 ± 0.70	33.02 ± 1.65	9.6 ± 0.28	13.3 ± 0.67	5.5 ± 0.07	4.9 ± 0.15
	Fe(NH_4_)_2_(SO_4_)_2_	37.1 ± 1.86	2.9 ± 0.09	10.19 ± 0.74	19.58 ± 0.98	4.1 ± 0.31	7.9 ± 0.40	7.4 ± 0.11	5.9 ± 0.18
	FeC_2_O_4_	72.3 ± 3.62	-	8.06 ± 0.85	-	3.2 ± 0.34	-	7.9 ± 0.85	-

**Table 3:** Self-healing efficiency of various poly(AA-co-AAm) hydrogels having varying monomer and salt content.

Modifier	Self-Healing Efficiency with Different Ratio of Monomers, %		
	7/3	1/1	3/7
Hydrogel			
Pure	0.00	18.88 ± 1.13	26.02 ± 1.56
NiCl_2_	31.41 ± 1.88	60.25 ± 3.61	20.28 ± 1.22
AlCl_3_	7.12 ± 0.43	23.85 ± 1.43	6.44 ± 0.39
FeCl_3_	5.36 ± 0.33	76.76 ± 4.61	17.32 ± 1.04
Fe(NH_4_)_2_(SO_4_)_2_	51.47 ± 3.09	65.45 ± 3.63	72.12 ± 4.33
PEG gel			
Pure	49.08 ± 2.94	27.24 ± 1.63	18.98 ± 1.14
NiCl_2_	48.54 ± 2.91	90.4 ± 5.42	24.62 ± 1.48
AlCl_3_	28.53 ± 1.71	46.14 ± 2.77	79.98 ± 4.88
FeCl_3_	54.74 ± 3.28	100 ± 7.82	66.24 ± 3.97
Fe(NH_4_)_2_(SO_4_)_2_	–	55.21 ± 3.31	100 ± 6.50

**Table 4:** Comparison of hysteresis areas for hydrogels and modified PEG-gels.

Material	NiCl_2_	AlCl_3_	FeCl_3_
Hydrogel	30.53 ± 17.09	1.51 ± 1.51	51.67 ± 2.35
PEG-gel	3.06 ± 2.70	0.87 ± 0.00 *	-

* Only one positive loop, in fact, hysteresis is not reproduced.

**Table 5:** Inter-cycle hysteresis area dynamics and variation coefficients for hydrogels and PEG-gels with modifications.

Material	Modifier	The Slope of the Graph	Coefficient of Variation	Interpretation
Hydrogels	NiCl_2_	−20.36	0.56	Rapid degradation, high variability
	AlCl_3_	−3.03	1.00	Abrupt attenuation, complete passivation
	FeCl_3_	+4.71	0.05	Area growth, an exceptionally stable process
PEG gels	NiCl_2_	−2.79	0.88	Slow degradation, unstable response
	AlCl_3_	–	–	A single loop, the trend is not defined
	FeCl_3_	–	–	There are no positive loops

**Table 6:** General variation of hysteresis area for modified hydrogels and PEG gels.

Modifier	Change in Hysteresis Area for Hydrogels	Change in Hysteresis Area for PEG Gels
NiCl_2_	−40.71	−5.59
AlCl_3_	−3.03	0.00
FeCl_3_	+4.71	0.00

**Table 7:** Relative change in storage modulus (ΔGₑ, %) for poly(AA-co-AAm) hydrogels having varying monomer and salt content.

Modifier	Monomer Ratio (AA/AAm)		
	7/3	1/1	3/7
	Relative Change in Storage Modulus (ΔGₑ, %)		
Pure	0	0	0
$Ni{Cl}_{2}$	11.44	14.08	−53.56
$Al{Cl}_{3}$	22.33	53.55	−22.57
$Fe{Cl}_{3}$	−37.81	−31.78	−68.61
$Fe{\left(NH_{4}\right)}_{2}{\left(SO_{4}\right)}_{2}$	−56.44	−50.39	−86.63
$FeC_{2}O_{4}$	−87.95	−89.02	−89.43

**Table 8:** Ranking of modifiers by versatility for improving the self-healing efficiency of gels.

Modifier	Advantages	Limitations
Fe(NH_4_)_2_(SO_4_)_2_	Consistently high efficiency across a wide range of formulations for both matrix types; reduces network density, increasing chain mobility	Tendency to phase separation in a polymer matrix
FeCl_3_	Record efficiency in PEG gel at 1/1 (~100%); moderate loosening of the network creates optimal conditions for reversible bonds	Less predictable in hydrogels with excess AAm
NiCl_2_	High efficiency in PEG gel at 1/1 (90.4%); stable ionic conductivity	Efficiency is highly dependent on the monomer ratio
AlCl_3_	Unique efficiency in PEG gel at 3/7 (79.98%) due to reversibility of bonds; maximum network reinforcement	Low efficiency in hydrogels due to the formation of rigid, poorly reversible nodes

**Table 9:** Composition of the reaction mixture for the synthesis of poly(AA-co-AAm) hydrogels with varying monomer and salt content.

Reagent	Ratio of Monomers		
	7/3	1/1	3/7
	Concentration, mol		
AA (neutralized and residual)	1	1	1
Water in solution	2.8420		
AAm	0.4670	1.0890	2.5420
MBA	0.0010	0.0020	0.0030
Water for synthesis	8.6860	13.2970	24.0560
TMED	0.0190	0.0270	0.0445
PSA	0.0097	0.0140	0.0226
NiCl_2_	0.0057	0.0080	0.0133
AlCl_3_	0.0123	0.0172	0.0287
FeCl_3_	0.0059	0.0083	0.0138
Fe(NH_4_)_2_(SO_4_)_2_	0.0059	0.0083	0.0138
FeC_2_O_4_	0.0059	0.0083	0.0138

## Data Availability

The data used in this study are available upon request.

## Abbreviations

The following abbreviations are used in this manuscript:

AA	Acrylic acid
AAm	Acrylamide
ATR	Attenuated total reflectance
DMA	Dynamic mechanical analysis
DSC	Differential scanning calorimetry
FTIR	Fourier-transform infrared spectroscopy
LVE-R	Linear viscoelastic range
MBA	N,N’-methylenebisacrylamide
PEG	Polyethylene glycol
P(AA-co-AAm)	Poly(acrylic acid-co-acrylamide)
PSA	Ammonium peroxydisulfate
TEMED	N,N,N’,N’-tetramethylethylenediamine
TGA	Thermogravimetric analysis
I-V characteristics	Current–voltage characteristics
